# Applications of Deep Learning to Neuro-Imaging Techniques

**DOI:** 10.3389/fneur.2019.00869

**Published:** 2019-08-14

**Authors:** Guangming Zhu, Bin Jiang, Liz Tong, Yuan Xie, Greg Zaharchuk, Max Wintermark

**Affiliations:** Neuroradiology Section, Department of Radiology, Stanford Healthcare, Stanford, CA, United States

**Keywords:** artificial intelligence, deep learning, radiology, neuro-imaging, acquisition

## Abstract

Many clinical applications based on deep learning and pertaining to radiology have been proposed and studied in radiology for classification, risk assessment, segmentation tasks, diagnosis, prognosis, and even prediction of therapy responses. There are many other innovative applications of AI in various technical aspects of medical imaging, particularly applied to the acquisition of images, ranging from removing image artifacts, normalizing/harmonizing images, improving image quality, lowering radiation and contrast dose, and shortening the duration of imaging studies. This article will address this topic and will seek to present an overview of deep learning applied to neuroimaging techniques.

## Introduction

Artificial intelligence (AI) is a branch of computer science that encompasses machine learning, representation learning, and deep learning (1). A growing number of clinical applications based on machine learning or deep learning and pertaining to radiology have been proposed in radiology for classification, risk assessment, segmentation tasks, diagnosis, prognosis, and even prediction of therapy responses (2–10). Machine learning and deep learning have also been extensively used for brain image analysis to devise imaging-based diagnostic and classification systems of strokes, certain psychiatric disorders, epilepsy, neurodegenerative disorders, and demyelinating diseases (11–17).

**Figure 1:**
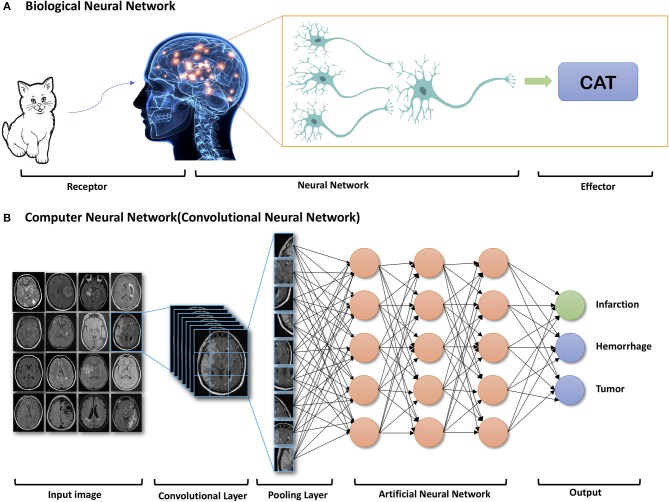
Example of components of Biologic Neural Network **(A)** and Computer Neural Network **(B)**. Reprinted with permission from Zaharchuk et al. (15). Copyright American Journal of Neuroradiology.

Recently, due to the optimization of algorithms, the improved computational hardware, and access to large amount of imaging data, deep learning has demonstrated indisputable superiority over the classic machine learning framework. Deep learning is a class of machine learning that uses artificial neural network architectures that bear resemblance to the structure of human cognitive functions (Figure 1). It is a type of representation learning in which the algorithm learns a composition of features that reflect a hierarchy of structures in the data (18). Convolutional neural networks (CNN) and recurrent neural networks (RNN) are different types of deep learning methods using artificial neural networks (ANN).

**Figure 2:**
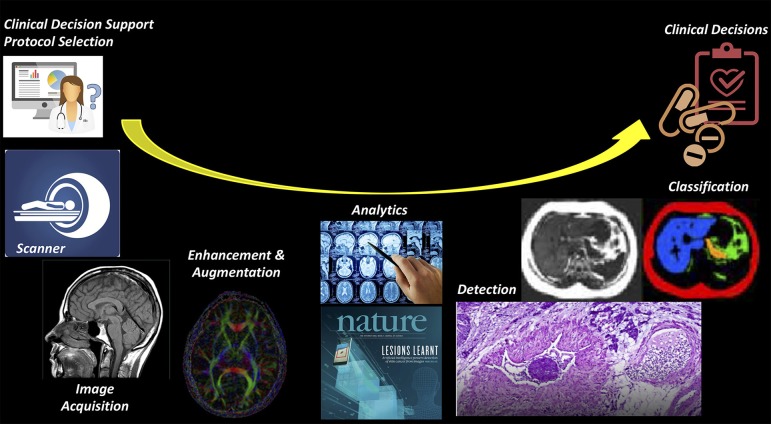
Imaging value chain. While most AI applications have focused on the downstream (or right) side of this pathway, such the use of AI to detect and classify lesions on imaging studies, it is likely that there will be earlier adoption for the tasks on the upstream (or left) side, where most of the costs of imaging are concentrated.

AI can be applied to a wide range of tasks faced by radiologists (Figure 2). Most initial deep learning applications in neuroradiology have focused on the “downstream” side: using computer vision techniques for detection and segmentation of anatomical structures and the detection of lesions, such as hemorrhage, stroke, lacunes, microbleeds, metastases, aneurysms, primary brain tumors, and white matter hyperintensities (6, 9, 15, 19). On the “upstream” side, we have just begun to realize that there are other innovative applications of AI in various technical aspects of medical imaging, particularly applied to the acquisition of images. A variety of methods for image generation and image enhancement using deep learning have recently been proposed, ranging from removing image artifacts, normalizing/harmonizing images, improving image quality, lowering radiation and contrast dose, and shortening the duration of imaging studies (8, 9, 15).

As RNNs are commonly utilized for speech and language tasks, the deep learning algorithms most applicable to radiology are CNNs, which can be efficiently applied to image segmentation and classification. Instead of using more than billions of weights to implement the full connections, CNNs can mimics mathematic operation of convolution, using convolutional and pooling layers (Figure 1) and significantly reduce the number of weights. CNNs can also allow for spatial invariance. For different convolutional layers, multiple kernels can be trained and then learn many location-invariant features. Since important features can be automatically learned, information extraction from images in advance of the learning process is not necessary. Therefore, CNNs are relatively easy to apply in clinical practice.

There are many challenges related to the acquisition and post-processing of neuroimages, including the risks of radiation exposure and contrast agent exposure, prolonged acquisition time, and image resolution. In addition, to expert parameter tuning of scanners always required to optimize reconstruction performance, especially in the presence of sensor non-idealities and noise (20). Deep learning has the opportunity to have a significant impact on such issues and challenges, with fewer ethical dilemmas and medical legal risks compared to applications for diagnosis and treatment decision making (21). Finally, these deep learning approaches will make imaging much more accessible, from many perspectives, including cost, patient safety, and patient satisfaction.

Published deep learning studies focused on improving medical imaging techniques are just beginning to enter the medical literature. A Pubmed search on computer-aided diagnosis in radiology, machine learning, and deep learning for the year 2018 yielded more than 5,000 articles. The number of publications addressing deep learning as applied to medical imaging techniques is a small fraction of this number. Although many studies are not focused on neuroimaging, their techniques can often be adapted for neuroimaging. This article will address this topic and will seek to present an overview of deep learning applied to neuroimaging techniques.

## Using Deep Learning to Reduce the Risk Associated With Image Acquisition

There are many risks associated with different image acquisitions, such as ionizing radiation exposure and side effect of contrast agents. Deep learning based optimizing acquisition parameters is crucial to achieve diagnostically acceptable image quality at the lowest possible radiation dose and/or contrast agent dose.

### MRI

**Figure 3:**
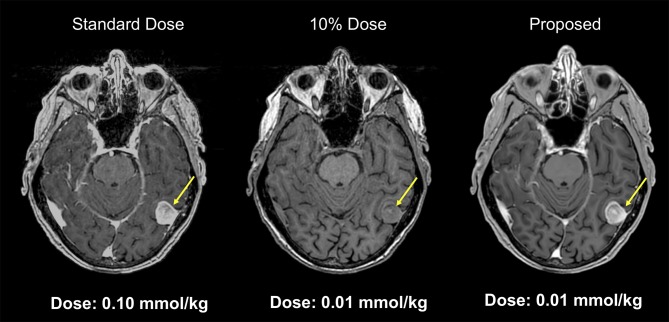
Example of low-dose contrast-enhaced MRI. Results from a deep network for predicting a 100% contrast dose image from a study obtained with 10% of the standard contrast dose. This example MRI is abtained from a patient with menigioma. Such methods may enable diagnostic quality images to be acquired more safely in a wider range of patients (Courtesy of Subtle Medical, Inc.).

Gadolinium-based contrast agents (GBCAs) have become indispensable in routine MR imaging. Though considered safe, CBCAs were linked with nephrogenic systemic fibrosis, which is a serious, debilitating, and sometimes life-threatening condition. There is ongoing discussion regarding the documented deposition of gadolinium contrast agents in body tissues including the brain, especially for those patients who need repeated contrast administration (22). Recent publications have reported the gadolinium deposition in the brain tissue, most notably in the dentate nuclei and globus pallidus (23, 24). This deposition can probably be minimized by limiting the dose of gadolinium used (25). Unfortunately, low-dose contrast-enhanced MRI is typically of insufficient diagnostic image quality. Gong et al. (26) implemented a deep learning model based on an encoder-decoder CNN to obtain diagnostic quality contrast-enhanced MRI with low-dose gadolinium contrast. In this study 60 patients with brain abnormalities received 10% low-dose preload (0.01 mmol/kg) of gadobenate dimeglumine, before perfusion MR imaging with full contrast dosage (0.1 mmol/kg). Pre-contrast MRI and low-dose post-contrast MRI of training set were introduced as inputs, and full dose post-contrast MRI as Ground-truth. The contrast uptake in the low-dose CE-MRI is noisy, but does include contrast information. Through the training, the network learned the guided denoising of the noisy contrast uptake extracted from the difference signal between low-dose and zero-dose MRIs, and then combine them to synthesize a full-dose CE-MRI. The results demonstrated that the deep learning algorithm was able to extract diagnostic quality images with gadolinium doses 10-fold lower than those typically used (Figure 3).

### CT

Computed Tomography (CT) techniques are widely used in clinical practice and involve a radiation risk. For instance, the radiation dose associated with a head CT is the same as 200 chest X-rays, or the amount most people would be exposed to from natural sources over 7 years. CT acquisition parameters can be adjusted to reduce the radiation dose, including reducing kilovoltage peak (kVp), milliampere-seconds (mAs), gantry rotation time, and increasing acquisition pitch. However, all these approaches also reduce image quality. Since an insufficient number of photons in the projection domain can lead to excessive quantum noise, the balance between image quality and radiation dose is always a trade-off.

Various image denoising approaches for CT techniques have been developed. Iterative reconstruction has been used, but sparsely, in part due to significant computational costs, time delays between acquisition and reconstruction, and a suboptimal “waxy” appearance of the augmented images (27, 28). Traditional image processing methods to remove image noise are also limited, because CT data is subject to both non-stationary and non-Gaussian noise processes. Novel denoising algorithms based on deep learning have been studied intensively and showed impressive potential (29). For example, Xie et al. (30) used a deep learning method based on a GoogLeNet architecture to remove streak artifacts due to missing projections in sparse-view CT reconstruction. The artifacts from low dose CT imaging were studied by residual learning, and then subtracted from the sparse reconstructed image to recover a better image. These intensively reconstructed images are comparable to the full-view projection reconstructed images. Chen et al. (28, 31) applied a residual encoder-decoder CNN, which incorporated a deconvolution network with shortcut (“bypass”) connections into a CNN model, to reduce the noise level of CT images. The model learned a feature mapping from low- to normal-dose images. After the training, it achieved a competitive performance in both qualitative and quantitative aspects, while compared with other denoising methods. Kang (27) applied a CNN model using directional wavelets for low-dose CT reconstruction. Compared to model-based iterative reconstruction methods, this algorithm can remove complex noise patterns from CT images with greater denoising power and faster reconstruction time. Nishio et al. (32) trained auto-encoder CNN for pairs of standard-dose (300 mA) CT images and ultra-low-dose (10 mA) CT images, and then used the trained algorithm for patch-based image denoising of ultra-low-dose CT images. The study demonstrated the advantages of this method over block-matching 3D (BM3D) filtering for streak artifacts and other types of noise. Many other deep learning-based approaches have been proposed in radiation-restricted applications, such as adversarially trained networks, sharpness detection network, 3D dictionary learning, and discriminative prior-prior image constrained compressed sensing (33–36).

Reconstruction algorithms to denoise the output low-quality images or remove artifacts have been studied intensively (27, 28, 30–32). Gupta et al. (37) implemented a relaxed version of projected gradient descent with a CNN for sparse-view CT reconstruction. There is a significant improvement over total variation-based regularization and dictionary learning for both noiseless and noisy measurements. This framework can also be used for super-resolution, accelerated MRI, or deconvolution, etc. Yi et al. used adversarially trained network and sharp detection network to achieve sharpness-aware low-dose CT denoising (34).

Since matched low- and routine-dose CT image pairs are difficult to obtain in multiphase CT, Kang et al. (38) proposed a deep learning framework based on unsupervised learning technique to solve this problem. They applied a cycle-consistent adversarial denoising network to learn the mapping between low- and high-dose cardiac phases. Their network did not introduce artificial features in the output images.

### Sparse-Data CT

The reconstruction of Sparse-data CT always compromises structural details and suffers from notorious blocky artifacts. Chen et al. (39) implemented a Learned experts' assessment-based reconstruction network (LEARN) for sparse-data CT. The network was evaluated with Mayo Clinic's low-dose challenge image data set and was proved more effectively than other methods in terms of artifact reduction, feature preservation, and computational speed.

### PET

Radiation exposure is a common concern in PET imaging. To minimize this potential risk, efforts have been made to reduce the amount of radio-tracer usage in PET imaging. However, low-dose PET is inherently noisy and has poor image quality. Xiang et al. combined 4-fold reduced time duration 18F-fluorodeoxyglucose (FDG) PET images and co-registered T1-weighted MRI images to reconstruct standard dose PET (40). Since PET image quality is to a first degree linear with true coincidence events recorded by the camera, such a method could also be applied to reduced dose PET. Kaplan and Zhu (41) introduced a deep learning model consisting an estimator network and a generative adversarial network (GAN). After training with simulated 10x lower dose PET data, the networks reconstructed standard dose images, while preserving edge, structural, and textural details.

Using a simultaneous PET/MRI scanner, Xu et al. (42) proposed an encoder-decoder residual deep network with concatenate skip connections to reconstruct high quality brain FDG PET images in patients with glioblastoma multiforme using only 0.5% of normal dose of radioactive tracer. To take advantage of the higher contrast and resolution of the MR images, they also included T1-weighted and T2-FLAIR weighted images as inputs to the model. Furthermore, they employed a “2.5D” model in which adjacent slice information is used to improve the prediction of a central slice. These modifications significantly reduced noise, while robustly preserving resolution and detailed structures with comparable quality to normal-dose PET images.

**Figure 4:**
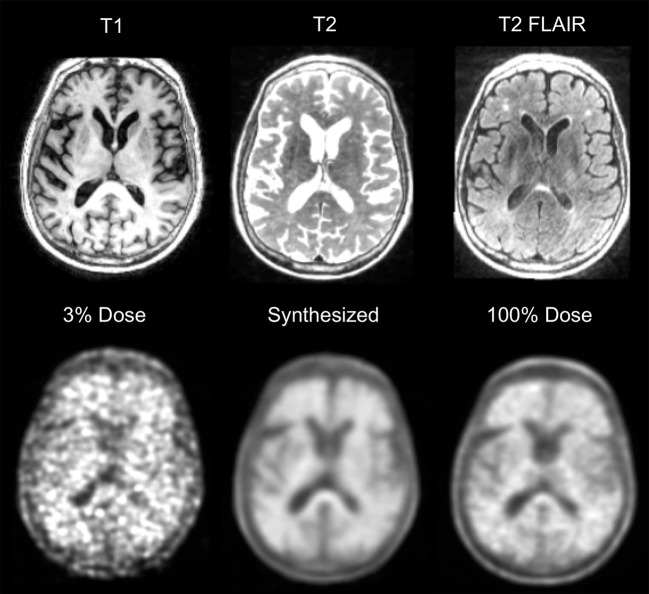
Example of ultra-low dose 18F-florbetaben PET/MRI. Example of a positive 18F-florbetaben PET/MRI study acquired at 0.24 mCi, ~3% of a standard dose. Similar image quality is present in the 100% dose image and the synthetized image, which was created using a deep neural network along with MRI information such as T1, T2, and T2-FLAIR. As Alzheimer Disease studies are moving toward cognitively normal and younger patients, reducing dose would be helpful. Furthermore, tracer costs could be reduced if doses can be shared.

These general principles were also applied by Chen et al. to simulated 1% dose 18F-florbetaben PET imaging (43). This amyloid tracer is used clinically in the setting of dementia of unknown origin. A “positive” amyloid study is compatible with the diagnosis of Alzheimer's disease, while a negative study essentially rules out the diagnosis (44, 45). Again, simultaneous PET/MRI was used to acquire co-registered contemporaneous T1-weighted and T2-FLAIR MR images, which were combined as input along with the 1% undersampled PET image. They showed the crucial benefit of including MR images in terms of retaining spatial resolution, which is critical for assessing amyloid scans. They found that clinical readers evaluating the synthesized full dose images did so with similar accuracy to their own intra-reader reproducibility. More recently, the same group has demonstrated that the trained model can be applied to true (i.e., not simulated) ultra-low dose diagnostic PET/MR images (Figure 4).

## Accelerate Imaging Acquisition and Reconstruct Under-sampled K-space

Image acquisition can be time-consuming. Reducing raw data samples or subsample k-space data can speed the acquisition, but result in suboptimal images. Deep learning based reconstruction methods can output good images from under-sampled datasets.

Compared to most other imaging modalities, MRI acquisition is substantially slower. The longer acquisition time limits the utility of MRI in emergency settings and often results in more motion artifact. It also contributes to its high cost. Acquisition time can be reduced by simply reducing the number of raw data samples. However, conventional reconstruction methods for these sparse data often produce suboptimal images. Newer reconstruction methods deploying deep learning have the ability to produce images with good quality from these under-sampled data acquired with shorter acquisition times (46). This approach has been applied in Diffusion Kurtosis Imaging (DKI) and Neurite Orientation Dispersion and Density Imaging (NODDI). DKI and NODDI are advanced diffusion sequences that can characterize tissue microstructure but require long acquisition time to obtain the required data points. Using a combination of q-Space deep learning and of simultaneous multi-slice imaging, Golkov et al. (47) were able to reconstruct DKI from only 12 data points and NODDI from only 8 data points, achieving an unprecedented 36-fold scan time reduction for quantitative diffusion MRI. These results suggest that there is considerable amount of information buried within the limited number of data points that can be retrieved with deep learning methods.

Another way to reduce acquisition time is to subsample k-space data. However, naive undersampling of k-space will cause aliasing artifact once the under-sampling rate exceeds the Nyquist conditions. Hyun et al. (48) trained a deep learning network, using pairs of subsampled and fully sampled k-space data as inputs and outputs respectively, to reconstruct images from sub-sampled data. They reinforced the subsampled k-space data with a few low-frequency k-space data to improve image contrast. Their network was able to generate diagnostic quality images from sampling only 29% of k-space.

Lee et al. (49) investigated deep residual networks to remove global artifacts from under-sampled k-space data. Deep residual networks are a special type of network that allows stacking of multiple layers to create a very deep network without degrading the accuracy of training. Compared to non-AI based fast-acquisition techniques such as compressed sensing MRI (which randomly sub-samples k-space) and parallel MRI (which uses multiple receiver coils), Lee's technique achieved better artifact reduction and use much shorter computation time.

Deep learning techniques for acceleration and reconstruction are not limited to static imaging, but are also applicable for dynamic imaging, such as cardiac MRI. Due to inherent redundancy within adjacent slices and repeated cycles in dynamic imaging, the combination of under-sampling and using Neural Networks for reconstruction seem to be the perfect solution. Schelmper's (50) trained CNN to learn the redundancies and the spatio-temporal correlations from 2D cardiac MR images. Their CNN outperformed traditional carefully handcrafted algorithms in terms of both reconstruction quality and speed. Similarly, Majumdar (51) address the problem of real-time dynamic MRI reconstruction by using a stacked denoising autoencoder. They produced superior images in shorter time, when compared to CS based technique and Kalman filtering techniques.

Hammernik et al. (52) introduced a variational network for accelerated Parallel Imaging-based MRI reconstruction. The reconstruction time was 193 ms on a single graphics card, and the MR images preserved the natural appearance as well as pathologies that were not included in the training data set. Chen et al. (53) also developed a deep learning reconstruction approach based on a variational network to improve the reconstruction speed and quality of highly undersampled variable-density single-shot fast spin-echo imaging. This approach enables reconstruction speeds of ~0.2 s per section, allowing a real-time image reconstruction for practical clinical deployment. This study showed improved image quality with higher perceived signal-to-noise ratio and improved sharpness, when compared with conventional parallel imaging and compressed sensing reconstruction. Yang et al. (54) proposed a deep architecture based on Alternating Direction Method of Multipliers algorithm (ADMM-Net) to optimize a compressed sensing-based MRI model. The results suggested high reconstruction accuracy with fast computational speed.

Several studies also used generative adversarial networks to model distributions (low-dimensional manifolds) and generating natural images (high-dimensional data) (35, 55). Mardani et al. (56) proposed a compressed sensing framework using generative adversarial networks (GAN) to model the low-dimensional manifold of high-quality MRI. This is combined with a compressed sensing framework, a method known as GANCS. It offers reconstruction times of under a few milliseconds and higher quality images with improved fine texture based on multiple reader studies.

## Artifacts Reduction

Image denoising is an important pre-processing step in medical image analysis, especially in low-dose techniques. Much research has been conducted on the subject of computer algorithms for image denoising for several decades, with varying success. Many attempts based on machine learning (57) or deep learning (58, 59) have been successfully implemented for denoising of medical images.

Standard reconstruction approaches involve approximating the inverse function with multiple *ad hoc* stages in a signal processing chain. They depend on the details of each acquisition strategy, and requires parameter tuning to optimize image quality. Zhu et al. (20) implemented a unified framework system called AUTOMAP, using a fully-connected deep neural network to reconstruct a variety of MRI acquisition strategies. This method is agnostic to the exact sampling strategy used, being trained on pairs of sensor data and ground truth images. They showed good performance for a wide range of k-space sampling methods, including Cartesian, spiral, and radial image acquisitions. The trained model also showed superior immunity to noise and reconstruction artifacts compared with conventional handcrafted methods. Manjón and Coupe (59) used two-stage strategy with deep learning for noise reduction. The first stage is to remove the noise using a CNN without estimation of local noise level present in the images. Then the filtered image is used as a guide image within a rotationally invariant non-local means filter. This approach showed competitive results for all the studied MRI acquisitions.

### Low Signal-To-Noise Ratio

MR images often suffers from low signal-to-noise ratio, such as DWI and 3D MR images. Jiang et al. (60) applied multi-channel feed-forward denoising CNNs, and Ran et al. (61) applied residual Encoder-Decoder wasserstein GAN, respectively, to restore the noise-free 3D MR images from the noisy ones.

**Figure 5:**
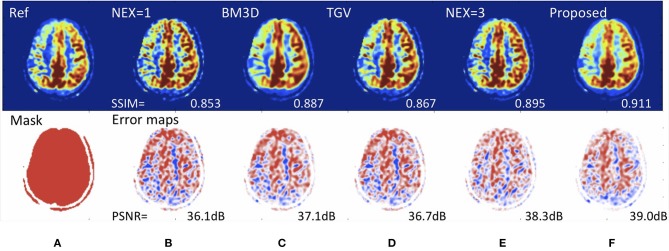
Deep learning for improving image quality of arterial spin labeling in a patient with right-sided Moyamoya disease. Reference scan **(A)** requiring 8 min to collect (nex = 6). Using a rapid scan acquired in 2 min (nex=1) **(B)**, it is possible to create an image **(F)** with the SNR of a study requiring over 4.5 min (nex = 3) **(E)**. The peak signal-to-noise (PSNR) performance is superior to existing de-noising methods such as **(C)** block matched 3D (BM3D) and **(D)** total generalized variation (TGV). Such methods could speed up MRI acquisition, enabling more functional imaging and perhaps reducing the cost of scanning.

Another MRI acquisition suffering from an inherently low-signal-to-noise ratio is arterial spin labeling (ASL) perfusion imaging. ASL has been used increasingly in neuroimaging because of its non-invasive and repeatable advantages in quantification and labeling. Repeated measurements of control/spin-labeled paired can lead to a fair image quality, but with the risk of motion artifacts. Ultas et al. (62) followed a mixed modeling approach including incorporting a Buxton kinetic model for CBF estimation, and training a deep fully CNN to learn a mapping from noisy image and its subtraction from the clean images. This approach produced high quality ASL images by denoising images without estimating its noise level. Due to a lower number of subtracted control/label pairs, this method also reduced ASL scan and reconstruction times, which makes ASL even more applicable in clinical protocols. Similarly, Kim et al. demonstrated image quality improvement using pseudocontinous ASL using data with 2 signal averages to predict images acquired with 6 signal averages, a roughly 3-fold speedup in imaging time (63). They also demonstrated that it was possible to reconstruct Hadamard-encoded ASL imaging from a subset of the reconstructed post-label delay images (though this does not allow for any speed-up in image acquisition). Owen et al. used a convolutional joint filter to exploit spatio-temporal properties of the ASL signal. This filter could reduce artifacts and improve the peak signal-to-noise ratio of ASL by up to 50% (64). Finally, Gong et al. demonstrated the benefits of including multi-contrast approaches (i.e., proton-density images along with ASL difference images) with multi-lateral guided filters and deep networks to boost the SNR and resolution of ASL (65). They also showed that the network could be trained with a relatively small number of studies and that it generalized to stroke patients (Figure 5).

### Spurious Noise

Proton MR spectroscopic imaging can measure endogenous metabolite concentration *in vivo*. The Cho/NAA ratio has been used to characterize brain tumors, such as glioblastoma. One challenge is the poor spectral quality, because of the artifacts caused by magnetic field inhomogeneities, subject movement, and improper water or lipid suppression. Gurbani et al. (66) applied a tiled CNN tuned by Bayesian optimization technique to analyze frequency-domain spectra to detect artifacts. This CNN algorithm achieved high sensitivity and specificity with an AUC of 0.951, while compared with the consensus decision of MRS experts. One particular type of MRS artifact is ghost or spurious echo artifact, due to insufficient spoiling gradient power. Kyathanahally et al. (67) implemented multiple deep learning algorithms, including fully connected neural networks, deep CNN, and stacked what-where auto encoders, to detect and correct spurious echo signals. After training on a large dataset with and without spurious echoes, the accuracy of the algorithm was almost 100%.

### Motion Artifact

MRI is susceptible to image artifacts, including motion artifacts due to the relatively long acquisition time. Küstner et al. (68) proposed a non-reference approach to automatically detect the presence of motion artifacts on MRI images. A CNN classifier was trained to assess the motion artifacts on a per-patch basis, and then used to localize and quantify the motion artifacts on a test data set. The accuracy of motion detection reached 97/100% in the head and 75/100% in the abdomen. There are several other studies on the detection or reducing of motion artifacts (69–71). Automating the process of motion detection can lead to more efficient scanner use, where corrupted images are re-acquired without relying on the subjective judgement of technologists.

### Metal Artifact

Artifacts resulting from metallic objects have been a persistent problem in computed tomography (CT) images over the last four decades. Gjesteby et al. (72) combined a CNN with the NMAR algorithm to reduce metal streaks in critical image regions. The strategy is able to map metal-corrupted images to artifact-free monoenergetic images.

### Crosstalk Noise

Attenuation correction is a critical procedure in PET imaging for accurate quantification of radiotracer distribution. For PET/CT, the attenuation coefficients (μ) are derived from the CT Hounsfield units from the CT portion of the examination. For PET/MRI, attenuation coefficient (μ) has been estimated from segmentation- and atlas-based algorithms. Maximum-likelihood reconstruction of activity and attenuation (MLAA) is a new method for generating activity images. It can produce attenuation coefficients simultaneously from emission data only, without the need of a concurrent CT or MRI. However, MLAA suffers from crosstalk artifacts. Hwang et al. (73) tested three different CNN architectures, such as convolutional autoencoder (CAE), U-net, and hybrid of CAE to mitigate the crosstalk problem in the MLAA reconstruction. Their CNNs generated less noisy and more uniform μ-maps. The CNNs also better resolved the air cavities, bones, and even the crosstalk problem.

Other studies have used deep learning to create CT-like images from MRI, often but not always for the purposes of PET/MRI attenuation correction. Nie et al. (74) applied an auto-context model to implement a context-aware deep convolutional GAN. It can generate a target image from a source image, demonstrating its use in predicting head CT images from T1-weighted MR images. This CT could be used for radiation planning or attenuation correction. Han (75) proposed a deep CNN with 27 convolutional layers interleaved with pooling and unpooling layers. Similar to Nie et al., the network was trained to learn a direct end-to-end mapping from MR images to their corresponding CTs. This method produced accurate synthetic CT results in near real time (9 s) from conventional, single-sequence MR images. Other deep learning networks, such as deep embedding CNN by Xiang et al. (76), Dixon-VIBE deep learning by Torrado-Carvajal et al. (77), GAN with two synthesis CNNs and two discriminator CNNs by Wolterink et al. (78), as well as deep CNN based on U-net architecture by Leynes et al. (79) and Roy et al. (80), were also proposed to generate pseudo CT from MRI.

Liu et al tried to train a network to transform T1-weighted head images into “pseudo-CT” images, which could be used for attenuate calculations (81). The errors in PET SUV could be reduced to less than 1% for most areas of the brain, about a 5-fold improvement over existing techniques such as atlas-based and 2-point Dixon methods. More recently, the same group has shown that it is possible to take non-attenuation correction PET brain images and using attenuation corrected images as the ground truth, to directly predict one from the other, without the need to calculate an attenuation map (82). This latter method could enable the development of new PET scanners that do not require either CT or MR imaging to be acquired, and which might be cheaper to site and operate.

### Random Noise

Medical fluoroscopy video is also sensitive to noise. Angiography is one medical procedure using live video, and the video quality is highly important. Speed is the main limitation of conventional denoising algorithms such as BM3D. Praneeth Sadda et al. (83) applied a deep neural network to remove Gaussian noise, speckle noise, salt and pepper noise from fluoroscopy images. The final output live video could meet and even exceed the efficacy of BM3D with a 20-fold speedup.

## Synthetic Image Production

Each imaging modality (X-ray, CT, MRI, ultrasound) as well as different MR sequences have different contrast and noise mechanisms and hence captures different characteristics of the underlying anatomy. The intensity transformation between any two modalities/sequences is highly non-linear. For example, Vemulapalli et al. (84) used a deep network to predict T1 images from T2 images. With deep learning, medical image synthesis can produce images of a desired modality without preforming an actual scan, such as creating CT images from MRI data. This can be of benefit because radiation can be avoided.

Ben-Cohen et al. (85) explored the use of full CNN and conditional GAN to reconstruct PET images from CT images. The deep learning system was tested for detection of malignant tumors in the live region. The results suggested a true positive ratio of 92.3% (24/26) and false positive ratio of 25% (2/8). This is surprising because no metabolic activity is expected to be present on CT images. It must be assumed that the CT features somehow contain information about tumor metabolism. In a reverse strategy, Choi and Lee (86) generated structural MR images from amyloid PET images using generative adversarial networks. Finally, Li et al. (87) used a 3D CNN architecture to predict missing PET data from MRI, using the ADNI study, and found it to be a better way of estimating missing data than currently existing methods.

### High-Field MRI

**Figure 6:**
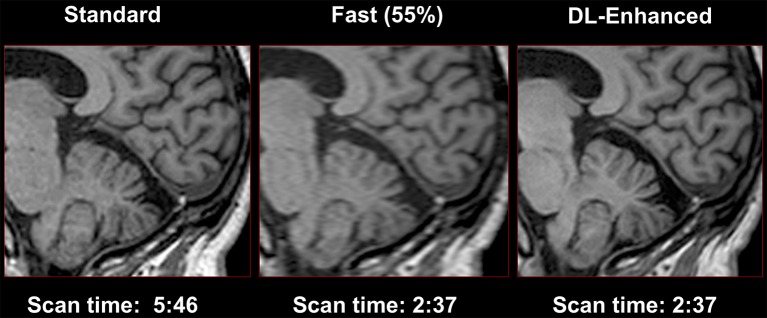
Use of convolutional neural networks to perform super-resolution. High-resolution T1-weighted imaging often requires long scan times to acquire sufficient resolution to resolve the gray-white border and to estimate cortical thickness. Shorter scans may be obtained with lower resolution, and AI can be used to restore the required high resolution (Image courtesy of Subtle Medical Inc.).

More recently, AI based methods, such as deep CNN's, can take a low-resolution image as the input and then output a high-resolution image (88), with three operations, “patch extraction and representation,” “non-linear mapping,” and “reconstruction” (89). Higher (or super-) resolution MRI can be implemented using MRI scanners with higher magnetic field, such as advanced 7-T MRI scanners, which involves much higher instrumentation and operational costs. As an alternative, many studies have attempted to achieve super-resolution MRI images from low-resolution MRI images. Bahrami et al. (90) trained a deep learning architecture based CNN, inputting the appearance and anatomical features of 3T MRI images and outputting as the corresponding 7T MRI patch to reconstruct 7T-like MRI images. Lyu et al. (91) adapted two neural networks based on deep learning, conveying path-based convolutional encoder-decoder with VGG (GAN-CPCE) and GAN constrained by the identical, residual, and cycle learning ensemble (GAN-CIRCLE), for super-resolution MRI from low-resolution MRI. Both neural networks had a 2-fold resolution improvement. Chaudhari et al. (92) implemented a 3-D CNN entitled DeepResolve to learn residual-based transformations between high-resolution and lower-resolution thick-slice images of musculoskeletal MRI. This algorithm can maintain the resolution as diagnostic image quality with a 3-fold down-sampling. Similar methods have recently been applied to T1-weighted brain imaging, which requires a long acquisition time to obtain adequate resolution for cortical thickness mapping (Figure 6).

### Synthetic FLAIR

Synthetic MRI imaging has become more and more clinically feasible, but synthetic FLAIR images are usually of lower quality than conventional FLAIR images (93). Using conventional FLAIR images as target, Hagiwara et al. (94) applied a conditional GAN to generate improved FLAIR images from raw synthetic MRI imaging data. This work created improved synthetic FLAIR imaging with reduced swelling artifacts and granular artifacts in the CSF, while preserving lesion contrast. More recently, Wang et al. showed that improvements in image quality for all synthetic MR sequences could be obtained using a single model for multi-contrast synthesis along with a GAN discriminator, which was dubbed “OneforAll” (95). This offered superior performance to a standard U-net architecture trained on only one image contrast at a time. Readers scored equivalent image quality between the deep learning-based images and the conventional MR sequences for all except proton-density images. The deep learning based T2 FLAIR images were superior to the conventional images, due to the inherent noise suppression aspects of the training process.

## Image Registration

Deformable image registration is critical in clinical studies. Image registration is necessary to establish accurate anatomical correspondences. Intensity-based feature selection methods are widely used in medical image registration, but do not guarantee the exact correspondence of anatomic sites. Hand-engineered features, such as Gabor filters and geometric moment invariants, are also widely used, but do not work well for all types of image data. Recently, many AI-based methods have been used to perform image registration. Deep learning may be more promising when compared to other learning-based methods, because it does not require prior knowledge or hand-crafted features. It uses a hierarchical deep architecture to infer complex non-linear relationships quickly and efficiently (96).

Wu et al. (96) applied a convolutional stacked auto-encoder to identify compact and highly discriminative features in observed imaging data. They used a stacked two-layer CNN to directly learn the hierarchical basis filters from a number of image patches on the MR brain images. Then the coefficients can be applied as the morphological signature for correspondence detection to achieve promising registration results (97). Registration for 2D/3D image is one of the keys to enable image-guided procedures, including advanced image-guided radiation therapy. Slow computation and small capture range, which is defined as the distance at which 10% of the registrations fail, are the two major limitations of existing intensity-based 2D/3D registration approaches. Miao et al. (98) proposed a CNN regression approach, referred to as Pose Estimation via Hierarchical Learning (PEHL), to achieve real-time 2D/3D registration with large capture range and high accuracy. Their results showed an increased capture range of 99–306% and a success rate of 5–27.8%. The running time was ~0.1 s, about one tenth of the time consumption other intensity-based methods have. This CNN regression approach achieved significantly higher computational efficiency such that it is capable of real-time 2D/3D registration. Neylon et al. (99) presented a method based on deep neural network for automated quantification of deformable image registration. This neural network was able to quantify deformable image registration error to within a single voxel for 95% of the sub-volumes examined. Other studies also include fast predictive image registration with deep encoder-decoder network based on a Large Deformation Diffeomorphic Metric Mapping model (100).

## Quality Analysis

Quality control is crucial for accurate medical imaging measurement. However, it is a time-consuming process. Deep learning-based automatic assessment may be more objective and efficient. Lee et al. (101) applied a CNN to predict whether CT scans meet the minimal image quality threshold for diagnosis. Due to the relatively small number of cases, this deep learning network had a fair performance with an accuracy of 0.76 and an AUC of 0.78. Wu et al. (102) designed a computerized fetal ultrasound quality assessment (FUIQA) scheme with two deep CNNs (L-CNN and C-CNN). The L-CNN finds the region of interest, while the C-CNN evaluates the image quality.

## Challenges of Deep Learning Applied to Neuroimaging Techniques

In summary, deep learning is a machine learning method based on artificial neural networks (ANN), and encompasses supervised, unsupervised, and semi-supervised learning. Despite the promises made by many studies, reliable application of deep learning for neuroimaging still remains in its infancy and many challenges remain.

First of them is overfitting. Training a complex classifier with a small dataset always carries the risk of overfitting. Deep learning models tend to fit the data exceptionally well, but it doesn't mean that they generalize well. There are many studies that used different strategies to reduce overfitting, including regularization (103), early stopping (104), and drop out (105). While overfitting can be evaluated by performance of the algorithm on a separate test data set, the algorithm may not perform well on similar images acquired in different centers, on different scanners, or with different patient demographics. Larger data sets from different centers are typically acquired in different ways using different scanners and protocols, with subtly different image features, leading to poor performance (21). According to those, data augmentation without standard criteria cannot appropriately address issues encountered with small datasets. Overcoming this problem, known as “brittle AI,” is an important area of research if these methods are to be used widely. Deep learning is also an intensely data hungry technology. It requires a very large number of well labeled examples to achieve accurate classification and validate its performance for clinical implementation. Because upstream applications such as image quality improvement are essentially learning from many predictions in each image, this means that the requirements for large datasets are not as severe as for classification algorithms (where only one learning data point is available per person). Nonetheless, building large, public, labeled medical image datasets is important, while privacy concerns, costs, assessment of ground truth, and the accuracy of the labels remain stumbling blocks (18). One advantage of image acquisition applications is that the data is in some sense already labeled, with the fully sampled or high dose images playing the role of labels in classification tasks. Besides the ethical and legal challenges, the difficulty of physiologically or mechanistically interpreting the results of deep learning are unsettling to some. Deep networks are “black boxes” where data is input and an output prediction, whether classification or image, is produced (106). All deep learning algorithms operate in higher dimensions than what can be directly visualized by the human mind, which has been coined as “The Mythos of Model Interpretability” (107). Some estimates of the network uncertainly in prediction would be helpful to better interpret the images produced.

## Conclusion

Although deep learning techniques in medical imaging are still in their initial stages, they have been enthusiastically applied to imaging techniques with many inspired advancements. Deep learning algorithms have revolutionized computer vision research and driven advances in the analysis of radiologic images. Upstream applications to image quality and value improvement are just beginning to enter into the consciousness of radiologists, and will have a big impact on making imaging faster, safer, and more accessible for our patients.

## Author Contributions

GuZ: drafting the review. BJ, LT, YX, and GrZ: revising the review. MW: conception and design and revising the review.

### Conflict of Interest Statement

The authors declare that the research was conducted in the absence of any commercial or financial relationships that could be construed as a potential conflict of interest.

## References

[1] Jiang F Jiang Y Zhi H Dong Y Li H Ma S. Artificial intelligence in healthcare: past, present and future. Stroke Vasc Neurol. (2017) 2:230–43. 10.1136/svn-2017-000101 29507784 PMC5829945

[2] Mayo RC Leung J. Artificial intelligence and deep learning – Radiology's next frontier? Clin Imaging. (2018) 49:87–8. 10.1016/j.clinimag.2017.11.007 29161580

[3] Liew C. The future of radiology augmented with Artificial Intelligence: a strategy for success. Eur J Radiol. (2018) 102:152–6. 10.1016/j.ejrad.2018.03.019 29685530

[4] Choy G Khalilzadeh O Michalski M Do S Samir AE Pianykh OS. Current applications and future impact of machine learning in radiology. Radiology. (2018) 288:318–28. 10.1148/radiol.2018171820 29944078 PMC6542626

[5] Nichols JA Herbert Chan HW Baker MAB. Machine learning: applications of artificial intelligence to imaging and diagnosis. Biophys Rev. (2018) 11:111–8. 10.1007/s12551-018-0449-9 30182201 PMC6381354

[6] Savadjiev P Chong J Dohan A Vakalopoulou M Reinhold C Paragios N. Demystification of AI-driven medical image interpretation: past, present and future. Eur Radiol. (2018) 29:1616–24. 10.1007/s00330-018-5674-x 30105410

[7] Giger ML. Machine learning in medical imaging. J Am Coll Radiol. (2018) 15:512–20. 10.1016/j.jacr.2017.12.028 29398494

[8] Hosny A Parmar C Quackenbush J Schwartz LH Aerts HJWL. Artificial intelligence in radiology. Nat Rev Cancer. (2018) 18:500–10. 10.1038/s41568-018-0016-5 29777175 PMC6268174

[9] McBee MP Awan OA Colucci AT Ghobadi CW Kadom N Kansagra AP. Deep learning in radiology. Acad Radiol. (2018) 25:1472–80. 10.1016/j.acra.2018.02.018 29606338

[10] Fazal MI Patel ME Tye J Gupta Y. The past, present and future role of artificial intelligence in imaging. Eur J Radiol. (2018) 105:246–50. 10.1016/j.ejrad.2018.06.020 30017288

[11] Kamal H Lopez V Sheth SA. Machine learning in acute ischemic stroke neuroimaging. Front Neurol. (2018) 9:945. 10.3389/fneur.2018.00945 30467491 PMC6236025

[12] Mateos-Pérez JM Dadar M Lacalle-Aurioles M Iturria-Medina Y Zeighami Y Evans AC. Structural neuroimaging as clinical predictor: a review of machine learning applications. Neuroimage Clin. (2018) 20:506–22. 10.1016/j.nicl.2018.08.019 30167371 PMC6108077

[13] Feng R Badgeley M Mocco J Oermann EK. Deep learning guided stroke management: a review of clinical applications. J Neurointerv Surg. (2018) 10:358–62. 10.1136/neurintsurg-2017-013355 28954825

[14] Davatzikos C. Machine learning in neuroimaging: progress and challenges. Neuroimage. (2018) 197:652–6. 10.1016/j.neuroimage.2018.10.003 30296563 PMC6499712

[15] Zaharchuk G Gong E Wintermark M Rubin D Langlotz CP. Deep learning in neuroradiology. Am J Neuroradiol. (2018) 39:1776–84. 10.3174/ajnr.A5543 29419402 PMC7410723

[16] Middlebrooks EH Ver Hoef L Szaflarski JP. Neuroimaging in epilepsy. Curr Neurol Neurosci Rep. (2017) 17:32. 10.1007/s11910-017-0746-x 28324301

[17] Plis SM Hjelm DR Salakhutdinov R Allen EA Bockholt HJ Long JD. Deep learning for neuroimaging: a validation study. Front Neurosci. (2014) 8:229. 10.3389/fnins.2014.00229 25191215 PMC4138493

[18] Chartrand G Cheng PM Vorontsov E Drozdzal M Turcotte S Pal CJ. Deep learning: a primer for radiologists. RadioGraphics. (2017) 37:2113–31. 10.1148/rg.2017170077 29131760

[19] Tang A Tam R Cadrin-Chênevert A Guest W Chong J Barfett J. Canadian association of radiologists white paper on artificial intelligence in radiology. Can Assoc Radiol J. (2018) 69:120–35. 10.1016/j.carj.2018.02.002 29655580

[20] Zhu B Liu JZ Cauley SF Rosen BR Rosen MS. Image reconstruction by domain-transform manifold learning. Nature. (2018) 555:487–92. 10.1038/nature25988 29565357

[21] Pesapane F Volonté C Codari M Sardanelli F. Artificial intelligence as a medical device in radiology: ethical and regulatory issues in Europe and the United States. Insights Imaging. (2018) 9:745–53. 10.1007/s13244-018-0645-y 30112675 PMC6206380

[22] Ramalho J Ramalho M. Gadolinium deposition and chronic toxicity. Magn Reson Imaging Clin N Am. (2017) 25:765–78. 10.1016/j.mric.2017.06.007 28964466

[23] Gulani V Calamante F Shellock FG Kanal E Reeder SB. Gadolinium deposition in the brain: summary of evidence and recommendations. Lancet Neurol. (2017) 16:564–70. 10.1016/S1474-4422(17)30158-8 28653648

[24] Kanda T Nakai Y Oba H Toyoda K Kitajima K Furui S Gadolinium deposition in the brain. Magn Reson Imaging. (2016) 34:1346–50. 10.1016/j.mri.2016.08.024 27613998

[25] Khawaja AZ Cassidy DB Al Shakarchi J McGrogan DG Inston NG Jones RG. Revisiting the risks of MRI with Gadolinium based contrast agents—review of literature and guidelines. Insights Imaging. (2015) 6:553–8. 10.1007/s13244-015-0420-2 26253982 PMC4569598

[26] Gong E Pauly JM Wintermark M Zaharchuk G. Deep learning enables reduced gadolinium dose for contrast-enhanced brain MRI. J Magn Reson Imaging. (2018) 48:330–40. 10.1002/jmri.25970 29437269

[27] Kang E. A deep convolutional neural network using directional wavelets for low-dose X-ray ct reconstruction Eunhee. Med Phys. (2017) 44:1–32. 10.1002/mp.12344 29027238

[28] Chen H Zhang Y Zhang W Liao P Li K Zhou J. Low-dose CT via convolutional neural network. Biomed Opt Express. (2017) 8:679–94. 10.1364/BOE.8.000679 28270976 PMC5330597

[29] Zhang K Zuo W Chen Y Meng D Zhang L. Beyond a Gaussian denoiser: residual learning of deep CNN for image denoising. IEEE Trans Image Process. (2017) 26:3142–55. 10.1109/TIP.2017.2662206 28166495

[30] Xie S Zheng X Chen Y Xie L Liu J Zhang Y. Artifact removal using improved GoogLeNet for sparse-view CT reconstruction. Sci Rep. (2018) 8:1–9. 10.1038/s41598-018-25153-w 29712978 PMC5928081

[31] Chen H Zhang Y Kalra MK Lin F Chen Y Liao P Low-dose CT with a residual encoder-decoder convolutional neural network (RED-CNN). Clin Sci. (2017) 60:199–205. 10.1109/TMI.2017.2715284 PMC5727581 28622671

[32] Nishio M Nagashima C Hirabayashi S Ohnishi A Sasaki K Sagawa T. Convolutional auto-encoders for image denoising of ultra-low-dose CT. Heliyon. (2017) 3:e00393. 10.1016/j.heliyon.2017.e00393 28920094 PMC5577435

[33] Eck BL Fahmi R Brown KM Zabic S Raihani N Miao J. Computational and human observer image quality evaluation of low dose, knowledge-based CT iterative reconstruction. Med Phys. (2015) 42:6098–111. 10.1118/1.4929973 26429285 PMC4592430

[34] Yi X Babyn P. Sharpness-aware low-dose CT denoising using conditional generative adversarial network. J Digit Imaging. (2018) 31:655–69. 10.1007/s10278-018-0056-0 29464432 PMC6148809

[35] Wolterink JM Leiner T Viergever MA Isgum I. Generative adversarial networks for noise reduction in low-dose CT. IEEE Trans Med Imaging. (2017) 36:2536–45. 10.1109/TMI.2017.2708987 28574346

[36] Bai T Yan H Jia X Jiang S Wang G Mou X. Z-index parameterization for volumetric CT image reconstruction via 3-D dictionary learning. IEEE Trans Med Imaging. (2017) 36:2466–78. 10.1109/TMI.2017.2759819 28981411 PMC5732496

[37] Gupta H Jin KH Nguyen HQ McCann MT Unser M. CNN-based projected gradient descent for consistent CT image reconstruction. IEEE Trans Med Imaging. (2018) 37:1440–53. 10.1109/TMI.2018.2832656 29870372

[38] Kang E Koo HJ Yang DH Seo JB Ye JC. Cycle-consistent adversarial denoising network for multiphase coronary CT angiography. Med Phys. (2018) 46:550–62. 10.1002/mp.13284 30449055

[39] Chen H Zhang Y Chen Y Zhang J Zhang W Sun H. LEARN: learned experts' assessment-based reconstruction network for sparse-data CT. IEEE Trans Med Imaging. (2018) 37:1333–47. 10.1109/TMI.2018.2805692 29870363 PMC6019143

[40] Xiang L Qiao Y Nie D An L Lin W Wang Q. Deep auto-context convolutional neural networks for standard-dose PET image estimation from low-dose PET/MRI. Neurocomputing. (2017) 267:406–16. 10.1016/j.neucom.2017.06.048 29217875 PMC5714510

[41] Kaplan S Zhu YM. Full-dose PET image estimation from low-dose PET image using deep learning: a pilot study. J Digit Imaging. (2018) [Epub ahead of print]. 10.1007/s10278-018-0150-3 30402670 PMC6737135

[42] Xu J Gong E Pauly J Zaharchuk G 200x low-dose PET reconstruction using deep learning. (2017). arXiv[Preprint].arXiv:1712.04119. Available online at: https://arxiv.org/abs/1712.04119

[43] Chen KT Gong E de Carvalho Macruz FB Xu J Boumis A Khalighi M. Ultra–low-dose 18F-florbetaben amyloid PET imaging using deep learning with multi-contrast MRI inputs. Radiology. (2018) 290:649–56. 10.1148/radiol.2018180940 30526350 PMC6394782

[44] Sabri O Sabbagh MN Seibyl J Barthel H Akatsu H Ouchi Y. Florbetaben PET imaging to detect amyloid beta plaques in Alzheimer's disease: phase 3 study. Alzheimers Dement. (2015) 11:964–74. 10.1016/j.jalz.2015.02.004 25824567

[45] Villemagne VL Ong K Mulligan RS Holl G Pejoska S Jones G Amyloid imaging with 18F-florbetaben in Alzheimer disease and other dementias. J Nucl Med. (2011) 52:1210–7. 10.2967/jnumed.111.089730 21764791

[46] Wang S Su Z Ying L Peng X Zhu S Liang F Accelerating magnetic resonance imaging via deep learning. In: 2016 IEEE 13th International Symposium on Biomedical Imaging (ISBI). Prague: IEEE (2016). p. 514–7. 10.1109/ISBI.2016.7493320 PMC6839781 31709031

[47] Golkov V Dosovitskiy A Sperl JI Menzel MI Czisch M Samann P. q-Space deep learning: twelve-fold shorter and model-free diffusion MRI scans. IEEE Trans Med Imaging. (2016) 35:1344–51. 10.1109/TMI.2016.2551324 27071165

[48] Hyun CM Kim HP Lee SM Lee S Seo JK. Deep learning for undersampled MRI reconstruction. Phys Med Biol. (2018) 63:135007. 10.1088/1361-6560/aac71a 29787383

[49] Lee D Yoo J Tak S Ye JC. Deep residual learning for accelerated MRI using magnitude and phase networks. IEEE Trans Biomed Eng. (2018) 65:1985–95. 10.1109/TBME.2018.2821699 29993390

[50] Schlemper J Caballero J Hajnal J V Price AN Rueckert D. A Deep cascade of convolutional neural networks for dynamic MR image reconstruction. IEEE Trans Med Imaging. (2018) 37:491–503. 10.1109/TMI.2017.2760978 29035212

[51] Majumdar A Real-time dynamic mri reconstruction using stacked denoising autoencoder (2015). arXiv:1503.06383.

[52] Hammernik K Klatzer T Kobler E Recht MP Sodickson DK Pock T. Learning a variational network for reconstruction of accelerated MRI data. Magn Reson Med. (2018) 79:3055–71. 10.1002/mrm.26977 29115689 PMC5902683

[53] Chen F Taviani V Malkiel I Cheng JY Tamir JI Shaikh J. Variable-density single-shot fast spin-echo MRI with deep learning reconstruction by using variational networks. Radiology. (2018) 289:366–73. 10.1148/radiol.2018180445 30040039 PMC6209075

[54] Yang Y Sun J Li H Xu Z Deep ADMM-net for compressive sensing MRI. Advances in Neural Information Processing Systems (NIPS). Barcelona (2016). p. 10–18.

[55] Quan TM Nguyen-Duc T Jeong WK. Compressed sensing MRI Reconstruction using a generative adversarial network with a cyclic loss. IEEE Trans Med Imaging. (2018) 37:1488–97. 10.1109/TMI.2018.2820120 29870376

[56] Mardani M Gong E Cheng JY Vasanawala SS Zaharchuk G Xing L Deep generative adversarial neural networks for compressive sensing (GANCS) MRI. IEEE Trans Med Imaging. (2018) 38:167–79. 10.1109/TMI.2018.2858752 30040634 PMC6542360

[57] Kaur P Singh G Kaur P A review of denoising medical images using machine learning approaches. Curr Med Imaging Rev. (2017) 13:675–85. 10.2174/1573405613666170428154156 PMC6225344 30532667

[58] Gondara L Medical image denoising using convolutional denoising autoencoders. In: 2016 IEEE 16th International Conference on Data Mining Workshops (ICDMW). Barcelona (2016). p. 241–6. 10.1109/ICDMW.2016.0041

[59] Manjón J V Coupe P MRI denoising using deep learning. In: International Workshop on Patch-Based Techniques in Medical Imaging. Granada: Springer (2018). p. 12–9.

[60] Jiang D Dou W Vosters L Xu X Sun Y Tan T. Denoising of 3D magnetic resonance images with multi-channel residual learning of convolutional neural network. Jpn J Radiol. (2018) 36:566–74. 10.1007/s11604-018-0758-8 29982919

[61] Ran M Hu J Chen Y Chen H Sun H Zhou J Denoising of 3-D magnetic resonance images using a residual encoder-decoder wasserstein generative adversarial network. arXiv Prepr arXiv180803941 (2018). 10.1016/j.media.2019.05.001 31085444

[62] Ulas C Tetteh G Kaczmarz S Preibisch C Menze BH DeepASL: Kinetic model incorporated loss for denoising arterial spin labeled MRI via deep residual learning. In: Lect Notes Comput Sci (including Subser Lect Notes Artif Intell Lect Notes Bioinformatics). Granada (2018). 10.1007/978-3-030-00928-1_4

[63] Kim KH Choi SH Park S-H. Improving arterial spin labeling by using deep learning. Radiology. (2018) 287:658–66. 10.1148/radiol.2017171154 29267145

[64] Owen D Melbourne A Eaton-Rosen Z Thomas DL Marlow N Rohrer J Deep convolutional filtering for spatio-temporal denoising and artifact removal in arterial spin labelling MRI. In: International Conference on Medical Image Computing and Computer-Assisted Intervention. Granada: Springer (2018). p. 21–9.

[65] Gong E Pauly J Zaharchuk G Boosting SNR and/or resolution of arterial spin label (ASL) imaging using multi-contrast approaches with multi-lateral guided filter and deep networks. In: Proceedings of the Annual Meeting of the International Society for Magnetic Resonance in Medicine. Honolulu, HI (2017).

[66] Gurbani SS Schreibmann E Maudsley AA Cordova JS Soher BJ Poptani H. A convolutional neural network to filter artifacts in spectroscopic MRI. Magn Reson Med. (2018) 80:1765–75. 10.1002/mrm.27166 29520831 PMC6107370

[67] Kyathanahally SP Döring A Kreis R. Deep learning approaches for detection and removal of ghosting artifacts in MR spectroscopy. Magn Reson Med. (2018) 80:851–63. 10.1002/mrm.27096 29388313

[68] Küstner T Liebgott A Mauch L Martirosian P Bamberg F Nikolaou K. Automated reference-free detection of motion artifacts in magnetic resonance images. Magn Reson Mater Physics, Biol Med. (2018) 31:243–56. 10.1007/s10334-017-0650-z 28932991

[69] Tamada D Kromrey M-L Onishi H Motosugi U Method for motion artifact reduction using a convolutional neural network for dynamic contrast enhanced MRI of the liver. arXiv:1807.06956v1 (2018). p. 1–15. 10.2463/mrms.mp.2018-0156 PMC7067907 31061259

[70] Tamada D Onishi H Motosugi U Motion artifact reduction in abdominal MR imaging using the U-NET network. In: Proc ICMRM and Scientific Meeting of KSMRM, Paris (2018).

[71] Duffy BA Retrospective correction of motion artifact affected structural MRI images using deep learning of simulated motion. In: Med Imaging with Deep Learn (Midl 2018). London, UK (2018). p. 1–8.

[72] Gjesteby L Yang Q Xi Y Shan H Claus B Jin Y Deep learning methods for CT image- domain metal artifact reduction. In: Dev X-Ray Tomogr XI. (2017). p. 10391–31. Available online at: https://spie.org/Documents/ConferencesExhibitions/op17%20abstract.pdf#page=148.

[73] Hwang D Kim KY Kang SK Seo S Paeng JC Lee DS. Improving accuracy of simultaneously reconstructed activity and attenuation maps using deep learning. J Nucl Med. (2018) 59:1624–9. 10.2967/jnumed.117.202317 29449446

[74] Nie D Trullo R Lian J Wang L Petitjean C Ruan S. Medical image synthesis with deep convolutional adversarial networks. IEEE Trans Biomed Eng. (2018) 65:2720–30. 10.1109/TBME.2018.2814538 29993445 PMC6398343

[75] Han X. MR-based synthetic CT generation using a deep convolutional neural network method. Med Phys. (2017) 44:1408–19. 10.1002/mp.12155 28192624

[76] Xiang L Wang Q Nie D Zhang L Jin X Qiao Y. Deep embedding convolutional neural network for synthesizing CT image from T1-Weighted MR image. Med Image Anal. (2018) 47:31–44. 10.1016/j.media.2018.03.011 29674235 PMC6410565

[77] Torrado-Carvajal A Vera-Olmos J Izquierdo-Garcia D Catalano OA Morales MA Margolin J. Dixon-VIBE Deep Learning (DIVIDE) pseudo-CT synthesis for pelvis PET/MR attenuation correction. J Nucl Med. (2019) 60:429–35. 10.2967/jnumed.118.209288 30166357 PMC6910626

[78] Wolterink JM Dinkla AM Savenije MHF Seevinck PR van den Berg CAT Išgum I Deep MR to CT synthesis using unpaired data. In: International Workshop on Simulation and Synthesis in Medical Imaging. Québec City, QC: Springer (2017). p. 14–23.

[79] Leynes AP Yang J Wiesinger F Kaushik SS Shanbhag DD Seo Y Direct pseudoCT generation for pelvis PET/MRI attenuation correction using deep convolutional neural networks with multi-parametric MRI: zero echo-time and dixon deep pseudoCT (ZeDD-CT). J Nucl Med. (2017) 59:852–8. 10.2967/jnumed.117.198051 29084824 PMC5932530

[80] Roy S Butman JA Pham DL Synthesizing CT from ultrashort echo-time MR images via convolutional neural networks. In: International Workshop on Simulation and Synthesis in Medical Imaging. Québec City, QC: Springer (2017). p. 24–32.

[81] Liu F Jang H Kijowski R Bradshaw T McMillan AB. Deep learning MR imaging–based attenuation correction for PET/MR imaging. Radiology. (2018) 286:676–84. 10.1148/radiol.2017170700 28925823 PMC5790303

[82] Liu F Jang H Kijowski R Zhao G Bradshaw T McMillan AB. A deep learning approach for 18F-FDG PET attenuation correction. EJNMMI Phys. (2018) 5:24. 10.1186/s40658-018-0225-8 30417316 PMC6230542

[83] Sadda P Qarni T. Real-time medical video denoising with deep learning: application to angiography. Int J Appl Inf Syst. (2018) 12:22–28. 10.5120/ijais2018451755 29877510 PMC5985814

[84] Vemulapalli R Van Nguyen H Zhou SK Deep networks and mutual information maximization for cross-modal medical image synthesis. In: Deep Learning for Medical Image Analysis. Elsevier (2017). p. 381–403.

[85] Ben-Cohen A Klang E Raskin SP Amitai MM Greenspan H Virtual pet images from ct data using deep convolutional networks: initial results. In: International Workshop on Simulation and Synthesis in Medical Imaging. Québec City, QC: Springer (2017). p. 49–57.

[86] Choi H Lee DS. Generation of structural MR images from amyloid PET: application to MR-less quantification. J Nucl Med. (2018) 59:1111–7. 10.2967/jnumed.117.199414 29217736 PMC6910644

[87] Li R Zhang W Suk H-I Wang L Li J Shen D. Deep learning based imaging data completion for improved brain disease diagnosis. In: International Conference on Medical Image Computing and Computer-Assisted Intervention. Boston, MA: Springer (2014). p. 305–12. 10.1007/978-3-319-10443-0_39 PMC4464771 25320813

[88] Dong C Loy CC He K Tang X. Image super-resolution using deep convolutional networks. IEEE Trans Pattern Anal Mach Intell. (2016) 38:295–307. 10.1109/TPAMI.2015.2439281 26761735

[89] Higaki T Nakamura Y Tatsugami F Nakaura T Awai K. Improvement of image quality at CT and MRI using deep learning. Jpn J Radiol. (2018) 37:73–80. 10.1007/s11604-018-0796-2 30498876

[90] Bahrami K Shi F Rekik I Shen D Convolutional neural network for reconstruction of 7T-like images from 3T MRI using appearance and anatomical features. In: Deep Learning and Data Labeling for Medical Applications. Athens: Springer (2016). p. 39–47.

[91] Lyu Q You C Shan H Wang G Super-resolution MRI through Deep Learning. arXiv Prepr arXiv:181006776 (2018).

[92] Chaudhari AS Fang Z Kogan F Wood J Stevens KJ Gibbons EK. Super-resolution musculoskeletal MRI using deep learning. Magn Reson Med. (2018) 80:2139–54. 10.1002/mrm.27178 29582464 PMC6107420

[93] Campbell BC V Christensen S Parsons MW Churilov L Desmond PM Barber PA. Advanced imaging improves prediction of hemorrhage after stroke thrombolysis. Ann Neurol. (2013) 73:510–9. 10.1002/ana.23837 23444008 PMC3665631

[94] Hagiwara A Otsuka Y Hori M Tachibana Y Yokoyama K Fujita S. Improving the quality of synthetic FLAIR images with deep learning using a conditional generative adversarial network for pixel-by-pixel image translation. Am J Neuroradiol. (2018) 40:224–30. 10.3174/ajnr.A5927 30630834 PMC7028623

[95] Wang G OneforAll: improving synthetic MRI with multi-task deep learning using a generative model. In: ISMRM MR Value Workshop, Edinburgh, UK (2019).

[96] Wu G Kim M Wang Q Munsell BC Shen D. Scalable high-performance image registration framework by unsupervised deep feature representations learning. IEEE Trans Biomed Eng. (2016) 63:1505–16. 10.1109/TBME.2015.2496253 26552069 PMC4853306

[97] Wu G Kim M Wang Q Gao Y Liao S Shen D. Unsupervised deep feature learning for deformable registration of MR brain images. In: International Conference on Medical Image Computing and Computer-Assisted Intervention. Nagoya: Springer (2013). p. 649–56. 10.1007/978-3-642-40763-5_80 PMC4073478 24579196

[98] Miao S Wang ZJ Liao R A CNN Regression approach for real-time 2D/3D registration. IEEE Trans Med Imaging. (2016) 351352–63. 10.1109/TMI.2016.2521800 26829785

[99] Neylon J Min Y Low DA Santhanam A. A neural network approach for fast, automated quantification of DIR performance. Med Phys. (2017) 44:4126–38. 10.1002/mp.12321 28477340

[100] Yang X Kwitt R Styner M Niethammer M. Quicksilver: fast predictive image registration – A deep learning approach. Neuroimage. (2017) 158:378–96. 10.1586/14737175.2015.1028369 28705497 PMC6036629

[101] Lee JH Grant BR Chung JH Reiser I Giger M Assessment of diagnostic image quality of computed tomography (CT) images of the lung using deep learning. In: Medical Imaging 2018: Physics of Medical Imaging. Vol 10573 Houston, TX: International Society for Optics and Photonics (2018). p. 105731M.

[102] Wu L Cheng J-Z Li S Lei B Wang T Ni D. FUIQA: Fetal ultrasound image quality assessment with deep convolutional networks. IEEE Trans Cybern. (2017) 47:1336–49. 10.1109/TCYB.2017.2671898 28362600

[103] Kolbak M Lauria K Lee I Mohan S Phan HP Salisbury J Regularization for deep learning. In: Deep Learning. Cambridge: MIT Press (2016). p. 221–65.

[104] Prechelt L. Early stopping—but when? In: Neural Networks: Tricks of the Trade. Springer (2012). p. 53–67.

[105] Srivastava N Hinton G Krizhevsky A Sutskever I Salakhutdinov R Dropout: a simple way to prevent neural networks from overfitting. J Mach Learn Res. (2014) 15:1929–58.

[106] Litjens G Kooi T Bejnordi BE Setio AAA Ciompi F Ghafoorian M. A survey on deep learning in medical image analysis. Med Image Anal. (2017) 42:60–88. 10.1016/j.media.2017.07.005 28778026

[107] Lipton ZC The mythos of model interpretability. arXiv [Preprint]. arXiv:160603490. (2016).

